# Gender equity and career progression among Croatian oncology professionals compared with the ESMO global survey

**DOI:** 10.1186/s12913-025-13870-8

**Published:** 2025-12-10

**Authors:** Renata Kelemenic-Drazin, Anuska Budisavljevic, Natalija Dedic Plavetic, Tajana Silovski, Sanja Plestina, Vesna Telesmanic Dobric, Mario Nalbani, Marijana Jazvic, Iva Kardum Fucak, Mislav Conkas, Zvjezdana Boric-Mikez, Stjepko Plestina

**Affiliations:** 1 Department of Hematology, Oncology and Clinical Immunology, General Hospital Varazdin, I. Mestrovica 1, Varazdin, 42 000 Croatia; 2 Department of Hematology and Oncology, General Hospital Pula, Pula, Croatia; 3 Department of Oncology, University Hospital Centre Zagreb, Zagreb, Croatia; 4 Department of Rare Thoracic Tumors, Clinic for Pulmonary Diseases, University Hospital Centre Zagreb, Zagreb, Croatia; 5 Department of Oncology and Nuclear Medicine, General Hospital Zadar, Zadar, Croatia; 6 Department of Oncology, General Hospital Dubrovnik, Dubrovnik, Croatia; 7 Clinic for Oncology and Nuclear Medicine, Sestre Milosrdnice University Hospital Centre, Zagreb, Croatia; 8 Department of Gastroenterology, Hematology and Oncology, General Hospital Dr Tomislav Bardek Koprivnica, Koprivnica, Croatia; 9 Department of Medical Oncology and Hematology, County Hospital Cakovec, Cakovec, Croatia; 10 Department of Hematology and Oncology, General Hospital Dr Josip Bencevic Slavonski Brod, Slavonski Brod, Croatia

**Keywords:** Gender equity, Oncology career, Women in leadership, Career progression, Career advancement barriers

## Abstract

**Background:**

Based on the 2016 and 2021 ESMO Women for Oncology (W4O) surveys, which revealed persistent gender disparities in oncology, we conducted a national survey to examine career-related challenges among oncology professionals in Croatia and compare them with international trends.

**Methods:**

We administered an anonymous online survey adapted from the 2021 ESMO W4O questionnaire, contextualized for the Croatian setting. The survey explored the perceived impact of gender, political affiliation, religion, and sexual orientation on career development. Responses were analyzed by gender and age.

**Results:**

A total of 206 participants responded (74% women, 26% men), primarily medical and radiation oncologists (55%), followed by pathologists (15%) and surgical oncologists (5%). Among all respondents, 41% were aged ≤ 40, 18% were residents, and 43% had worked in oncology for less than 10 years. Most worked in university (42%) or general hospitals (39%). Gender was reported to moderately or significantly affect career progression in 40% of cases—more frequently than political affiliation (22%)—while religion and sexual orientation had minimal influence. Major gender-related barriers included poor work–life balance (69%), societal pressure (46%), unconscious bias (44%), and limited leadership opportunities for women (33%). Workplace gender-based discrimination was reported by 36%, and 38% had experienced or witnessed harassment; however, only 11% reported incidents. Although 80% valued career advancement, 34% were dissatisfied with their progression. Overall, 86% faced career barriers, primarily poor work–life balance (56%), lack of mentors (42%), and demotivating work environments (36%). Administrative overload was widespread: 59% felt overwhelmed, and 61% worked more than 8 h daily.

**Conclusions:**

Gender remains a substantial barrier to career advancement in oncology. Systemic interventions are needed to address work–life imbalance, discrimination, and structural burdens to support equity and sustainable career development.

**Supplementary Information:**

The online version contains supplementary material available at 10.1186/s12913-025-13870-8.

## Highlights

Croatian survey confirms poor work-life balance is the top barrier to oncology career progress.

Nearly 60% say administrative overload limits leadership roles and academic career growth.

Career dissatisfaction higher in Croatian oncologists, especially those aged 40 or younger.

Gender (40%) and political views (22%) remain key barriers, impacting women and young professionals.

Stronger support for gender quotas and reforms shows need for tailored national strategies.

**Supplementary Information:**

The online version contains supplementary material available at 10.1186/s12913-025-13870-8.

## Introduction

Persistent gender disparities across the health sector continue to shape professional trajectories, workplace dynamics, and leadership representation. Although women constitute most of the global health and care workforce - accounting for 67% according to the latest World Health Organization (WHO) data - only one in four holds a senior leadership position [1]. This structural imbalance limits not only women’s decision-making power but also the potential for more inclusive and representative leadership in healthcare systems [2, 3].

A growing body of research suggests that gender-balanced teams are associated with improved organizational outcomes, higher levels of collaboration, and, in clinical settings, potentially better patient care [4, 5] However, achieving true gender equity demands more than demographic shifts - it requires confronting deeply embedded social norms, addressing implicit bias, and ensuring equal access to mentorship, leadership development, and career progression [3, 6]. 

Within oncology, notable gains have been made in terms of female representation. For example, the European Society for Medical Oncology (ESMO) reported that the proportion of female members grew from 25% in 2004 to 49% in 2021 [7]. In recognition of these evolving demographics and the challenges they pose, ESMO established the *Women for Oncology* (W4O) initiative to monitor progress, raise awareness, and propose solutions to gender-based disparities in oncology careers. Surveys conducted by the W4O Committee in 2016 and 2021 highlighted multiple barriers experienced by women in oncology, including underrepresentation in leadership roles and gender-related obstacles to career development [8–11]. 

Building on these findings, and acknowledging the limited regional data available, we conducted a national survey of oncology professionals in Croatia to explore the perceived impact of gender and other personal factors - such as political affiliation, sexual orientation, and religion - on career progression. Our aim was to identify both overt and subtle barriers to professional development and to contribute context-specific data that may inform future strategies to promote gender equity and professional inclusion within oncology.

## Materials and methods

Building on the findings of the 2016 and 2021 *Women for Oncology* (W4O) surveys conducted by the European Society for Medical Oncology (ESMO), we carried out a national cross-sectional study among oncology professionals in Croatia from June to September 2024. The objective of this study was to identify career-related challenges faced by Croatian oncologists, compare them with previously published W4O data, and propose context-specific priorities and recommendations for addressing gender disparities in oncology.

The survey instrument was adapted from the W4O 2021 questionnaire, maintaining core components while introducing a contextual modification: political affiliation was included as a variable instead of ethnicity, given that non-Croatian nationals represent less than 1% of the population, according to the Croatian Bureau of Statistics [12]. The questionnaire consisted of seven sections: (i) demographics; (ii) household responsibilities; (iii) place of employment; (iv) barriers to career progression; (v) the perceived impact of diversity characteristics (gender, religion, sexual orientation, political affiliation) on professional development; (vi) inappropriate or discriminatory behaviour encountered in the workplace; and (vii) strategies for closing the gender gap.

The survey was administered via Google Forms and disseminated through Croatian national oncology societies, professional networks of multidisciplinary oncology teams, and social media channels. Participation was open to oncology professionals of all genders and specialties, working across various institutional settings in Croatia. All responses were collected anonymously.

Survey data were analysed according to respondent gender and age group (≤ 40 years vs. >40 years), consistent with ESMO membership categories. Categorical variables were compared using chi-square tests, while continuous variables were assessed with the Mann–Whitney U test. To account for the influence of age on gender differences, the Cochran–Mantel–Haenszel test was applied. Statistical significance was defined as a P value < 0.05. Analyses were performed using SAS software, version 9.4.

## Results

### Comparison of demographics and professional environment between our cohort and the ESMO global survey

Our study collected 206 responses, with a predominance of female respondents (74%) compared to 26% males, which is broadly consistent with the ESMO survey reporting 69% women and 30% men. Like the international cohort, a substantial proportion of respondents in our study were early-career professionals, with 41% aged ≤ 40 years, 18% trainees, and 43% practicing oncology for less than 10 years, aligning closely with the ESMO findings of 47% aged ≤ 40, 18% trainees, and 40% with less than 10 years of experience.

Regarding specialties, our cohort was primarily composed of medical oncologists and radiation oncologists (55%), followed by pathologists/cytologists (15%), surgical oncologists (5%), hemato-oncologists (4%), and radiologists (4%). The ESMO survey predominantly included medical oncologists (74%) and clinical/radiation oncologists (14%).

Workplace distribution was similar across both datasets, with respondents nearly equally split between clinical hospital centres (42% in our cohort vs. 44% in ESMO cohort) and general hospitals (39% vs. 44%, respectively). Clinical care occupied most of the working time in both studies, with 56% of our respondents dedicating over 50% of their time to clinical duties, while the ESMO cohort reported 60% clinical care time. However, our cohort reported higher engagement in research and teaching activities (37% allocating at least 10% of time) compared to 10% reported in the ESMO survey.

**Fig. 1:**
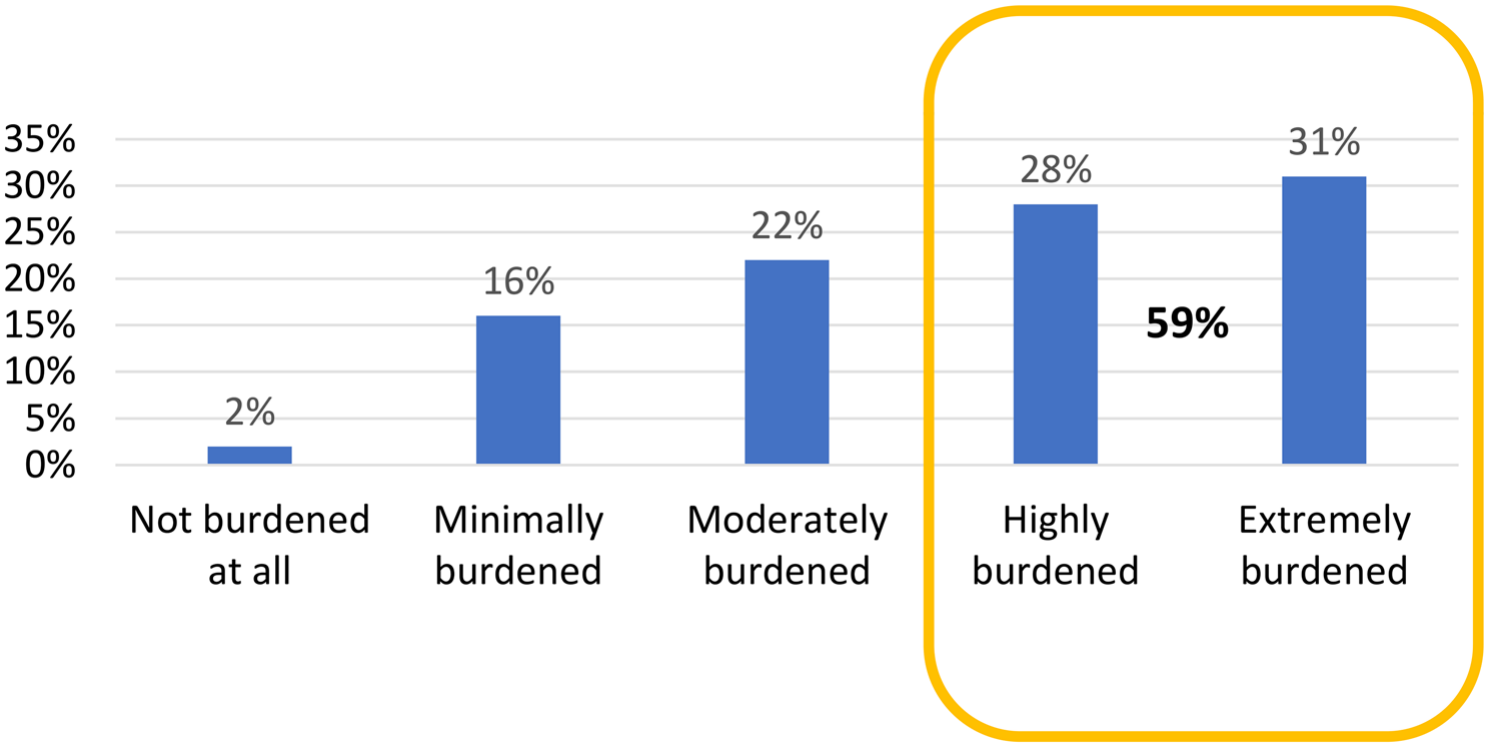
Administrative overload. Perceived administrative workload among oncology professionals, with most reporting a significant administrative burden

A notable difference was observed in administrative and managerial workload: 65% of our respondents reported spending 20–30% of their working time on administration, and 26% dedicated at least 10% to management tasks. Furthermore, 59% of respondents described administrative burden as significant, regardless of gender or age. (Fig. 1)

Most respondents (~ 80%), regardless of gender and age, believe that administrative tasks should not be the responsibility of oncologists but should be handled by administrative staff instead. Additionally, 35% of respondents advocated for protected time during working hours for administration, ranging from 4 to 8 h per week.

Workload intensity was underscored by the finding that 61% of our respondents worked more than eight hours daily, and only 14% refrained from working during weekends or days off, highlighting a considerable work-life imbalance.

In Croatia, as in the ESMO study, women remain underrepresented in leadership roles, with only 38% of respondents working in teams led by women (*p* = 0.022), compared to 39% reported in the ESMO data.

In summary, our local data reflect similar demographic and professional profiles as reported by the global ESMO survey, with differences in the extent of research involvement and administrative workload, emphasizing the need for institutional support to alleviate administrative burdens and promote sustainable working conditions for oncology professionals.

### Comparison of career progression challenges in oncology: ESMO survey vs. Croatian cohort

**Fig. 2:**
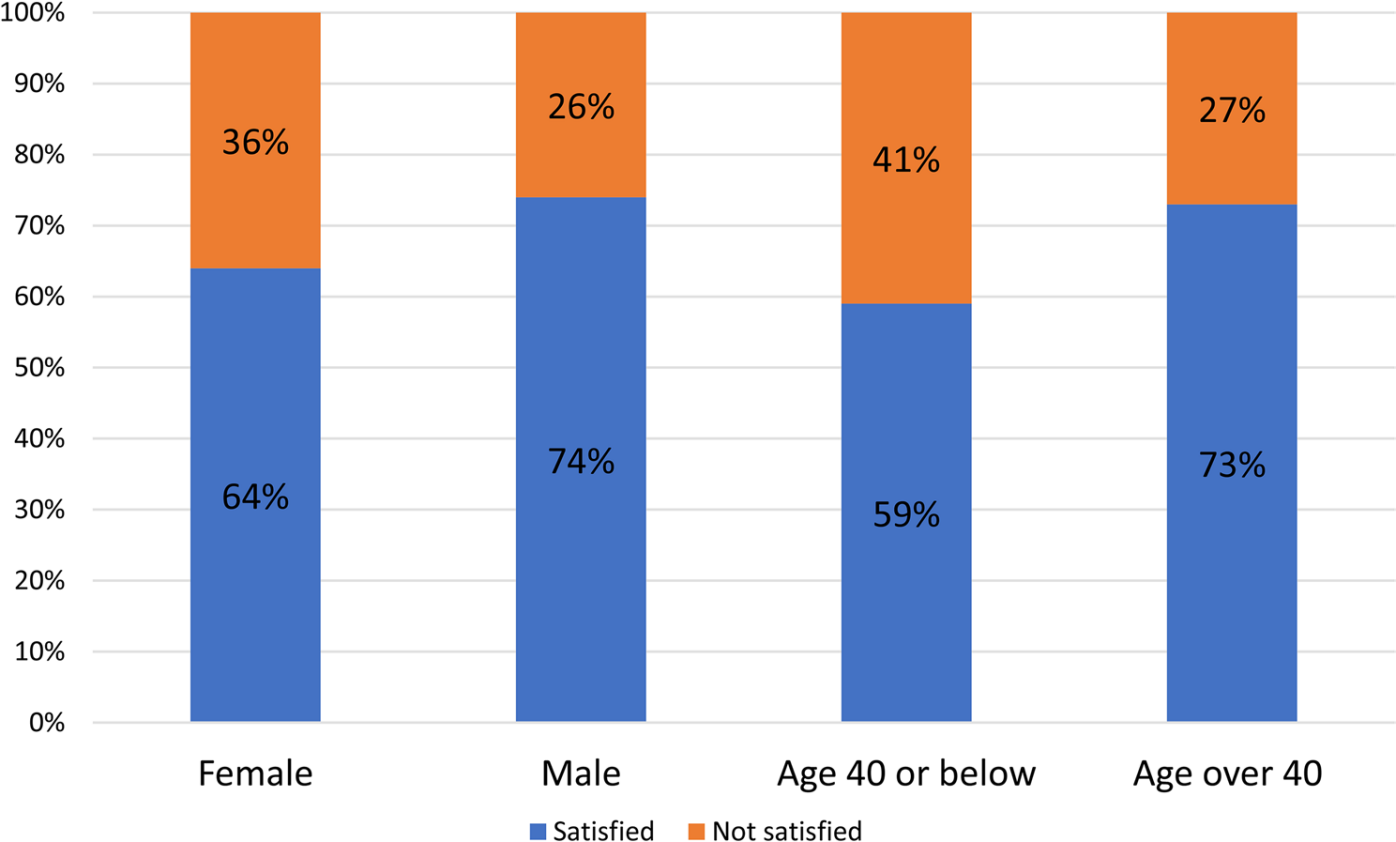
Career advancement satisfaction by gender and age, showing lower satisfaction levels among women and younger professionals in the Croatian survey

Career progression was deemed important by a large majority in both the global ESMO survey (90%) and the Croatian national cohort (80%). However, satisfaction with career advancement was lower in the Croatian sample, where 34% of respondents reported being only partially or not at all satisfied, compared to 28% in the ESMO survey. Women (36%) and younger professionals (≤ 40 years; 41%) were particularly dissatisfied, with comparable trends in the ESMO data (31% and 33%, respectively), suggesting heightened career progression challenges among these groups. (Fig. 2)

**Fig. 3:**
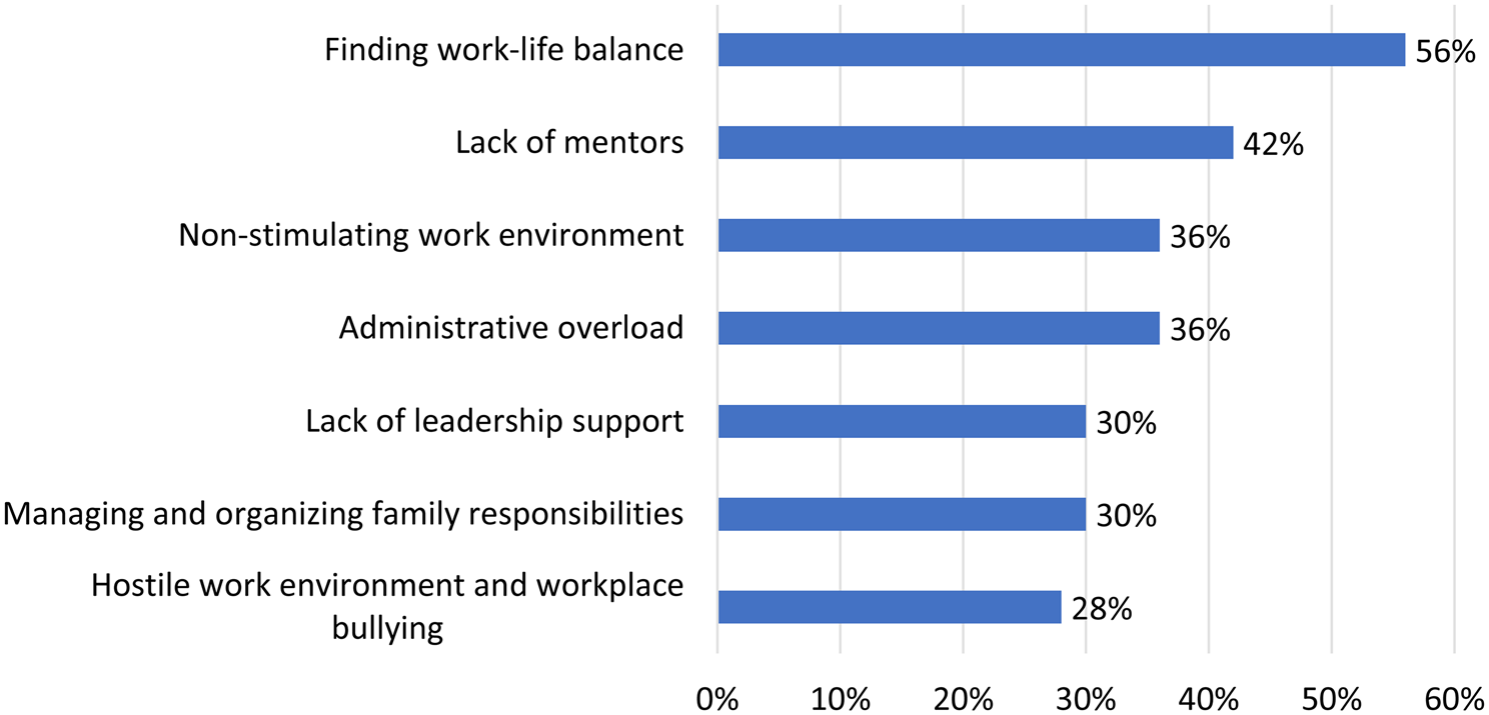
Main barriers to career advancement, with work–life balance, lack of mentorship, and unsupportive work environments most frequently reported by Croatian oncology professionals

Barriers to career advancement showed strong similarities across both datasets. In the ESMO survey, key obstacles included work-life balance (59.5%), lack of mentors or role models (39%), and family commitments (24%). Croatian respondents identified similar concerns: work-life balance (56%) and lack of mentorship (42%) were most frequently reported, followed by unsupportive work environments and administrative overload (36%), lack of supervisor support, and family-related challenges (30%). Notably, 28% of Croatian respondents reported experiencing hostile work environments or workplace bullying. (Fig. 3)

Significant age- and gender-specific barriers were identified. In the Croatian cohort, respondents aged ≤ 40 years cited mentorship deficits (*p* = 0.008), uninspiring work environments (*p* = 0.014), and financial limitations (*p* = 0.026) as major obstacles. Those > 40 years primarily reported lack of access to leadership roles (*p* = 0.022). Gender-based differences also emerged: men more often reported a lack of workplace stimulation (*p* = 0.042), while women identified gender bias, societal pressures related to gender stereotypes (*p* = 0.034), and challenges surrounding parental leave and reintegration (*p* = 0.021) as significant issues.

**Fig. 4:**
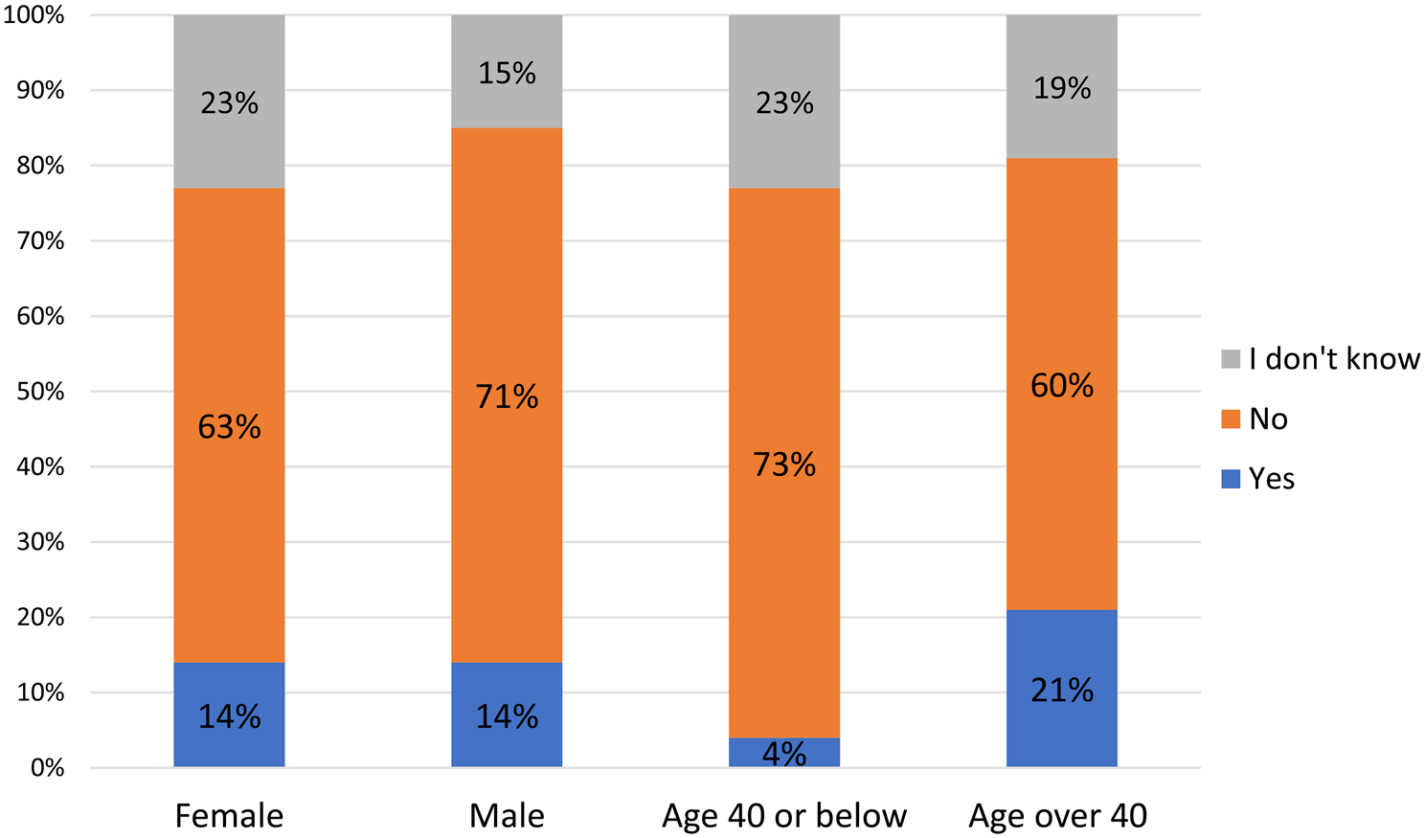
Unfulfilled professional potential among Croatian oncologists, with 63% of females and 71% of males reporting unmet career potential, particularly among younger professionals (73%)

A particularly concerning finding in the Croatian study was that 64% of respondents felt they had not fulfilled their potential as physicians, especially younger professionals (*p* = 0.008). (Fig. 4). Moreover, 83% believed that promotions were primarily influenced by managerial decisions, rather than experience (25%) or transparent hiring processes (14%).

Both datasets highlighted gender as the most influential identity-related barrier to career progression. In the ESMO survey, 25.5% of participants reported that gender had a significant impact on their careers, compared to much lower percentages for ethnicity (11%), sexual orientation (5%), and religion (3%). Gender was also linked to reduced opportunities (46%) and salary disparities (36%).

The Croatian data reflected similar but more pronounced patterns: 40% reported gender as a significant career factor, compared to political affiliation (22%), while religion and sexual orientation were largely considered irrelevant (< 10%). Notably, 56% of women and only 2% of men in Croatia reported reduced career opportunities due to gender (*p* < 0.0001). Comparable findings were reported in the ESMO data (60% of women vs. 19% of men; *p* < 0.0001), with the strongest effects again among younger professionals (52% vs. 44%, *p* = 0.0002).

Longitudinal trends indicate improvement: the proportion of ESMO respondents perceiving gender as a career barrier declined from 82% in 2016 to 66% in 2021, while the Croatian data from 2024 report 40%. Despite this, nearly half of respondents in both studies felt that little or no progress has been made in addressing gender inequality. In the ESMO data, this sentiment was especially strong among women over 40 (42.5% vs. 14% of men).

In conclusion, both global and national data consistently highlight persistent structural and gender-based barriers in oncology. The findings underscore the need for sustained, transparent, and targeted institutional efforts to ensure equity, inclusion, and professional fulfillment for all oncology professionals.

### Comparison of published ESMO data and our Croatian results on gender and discrimination in oncology

A comparison of our Croatian study and the published ESMO data reveals consistent gender-based disparities in oncology, despite regional differences in reporting rates. In the ESMO survey, 57% of women and 8% of men reported discrimination by a senior colleague, and 32% of women and 30% of men reported discrimination by a peer. In our study, 36% of participants experienced gender-based discrimination in the workplace, significantly more often among women (45%) than men (12%) (*p* < 0.001), with no significant difference by age group (≤ 40 years: 34% vs. >40 years: 36%, *p* = 0.727). Discrimination by patients was also prominent: in the ESMO cohort, 54% of women and 9% of men reported it, while in our study 43% of women and 9% of men did so (*p* < 0.001), with significantly higher rates among younger women (44% vs. 26% of men, *p* = 0.024).

Harassment was experienced by 38% of Croatian respondents, as mirroring ESMO data (38%), with women and younger professionals more affected, though subgroup differences in our cohort were not statistically significant. Reporting of harassment remained low in both studies, particularly in Croatia, where only 11% reported incidents compared to 22% in the ESMO study. The leading reason for non-reporting in our cohort was the belief that no action would be taken (61%), which is notably higher compared to the ESMO study (36%), followed by perceptions of low severity (27% vs. 28% ESMO data) and fear of retaliation (20% in both groups). The most reported incidents involved inappropriate sexist remarks (83% vs. 22% in the ESMO study).

These results indicate that, although overt misconduct may be less reported or recognized in Croatia, the gendered patterns of discrimination and the reluctance to report remain highly comparable. The findings highlight the urgent need for culturally tailored interventions, the implementation of transparent and trusted reporting mechanisms, and sustained institutional efforts to promote equity and psychological safety in oncology.

### Comparison of published and our study results on gender and family life impact on career

Published data show that women are significantly more likely than men to be primary caregivers for children and to perform household tasks, with differences evident in both younger (≤ 40 years) and older (> 40 years) groups. Our study confirms a higher childcare responsibility among younger women (52% vs. 32%, *p* = 0.032).

Published findings also report that parental leave and difficulties returning to work are greater obstacles for women, especially over 40. Similarly, our data indicate that career impacts parental leave more for older respondents (18% ≤40 years vs. 36% >40 years, *p* = 0.002) and affects childcare significantly more for younger women than men (52% vs. 32%, *p* = 0.032).

Social pressures and lack of family support are noted in published data as barriers for women’s career progression, which aligns with our findings that career significantly affects family, marriage, and social life, particularly in younger age groups regardless of gender.

In summary, both studies highlight the greater burden women face in balancing family and career, especially related to childcare and parental leave, with generational differences also apparent.

### Gender and pay gap

Published data show that significantly more women than men perceive a gender pay gap, especially among those over 40 years (36% women ≤ 40 vs. 13% men; 40.5% women > 40 vs. 9% men). Women also report that gender significantly impacts their career progression and pay disparities in oncology in their countries.

Although our study did not analyse individual salaries due to standardized pay scales in Croatian healthcare, where pay depends on qualifications rather than gender, the predominance of men in leadership roles suggests that pay inequality likely persists. Supporting this, data from Croatian Bureau of Statistics indicate that women in healthcare earn on average about 22% less than men, highlighting ongoing challenges regarding equal pay despite formal salary structures [13].

### Comparison of our national data with previously published ESMO survey results on gender equity in oncology

Our national survey results align with the previously published ESMO findings regarding the main barriers and facilitators of gender equity in oncology. In our study, the most reported barriers were lack of work–life balance (69%), societal pressures (46%), unconscious bias (44%), and lack of leadership development for women (33%), which closely mirrors the ESMO survey (54%, 32%, 34.5%, and 33%, respectively). A key distinction in our results is that women more frequently emphasized the importance of transparent career paths and salary structures (*p* = 0.029), a gender-related difference not observed in the ESMO study.

Our data also showed that flexible work is more strongly supported by respondents ≤ 40 years (46% vs. 27%, *p* = 0.006), consistent with the age-related trends reported in the ESMO paper.

In terms of facilitating gender equity, promoting work–life balance was the most supported measure in both surveys (68% vs. 57%). Development and leadership training (36% vs. 39%) and flexible working conditions (37% vs. 36%) were comparably supported. However, our respondents showed stronger support for implementing gender quotas in oncology committees and events (47% vs. 22%; *p* = 0.002) and for mentorship programmes for women (40% vs. 22%; *p* = 0.018), with these interventions significantly more supported by female respondents. There are no significant age-related differences in support for the programmes (all *p* > 0.05), although younger respondents show greater support for flexible educational programmes and family-friendly facilities at events.

Interestingly, both datasets indicate that a substantial proportion of oncology professionals perceive little or no progress in closing the gender gap since the beginning of their careers (39% in our study and 40% in the ESMO survey). In our study, age - unlike gender (*p* = 0.697) - emerged as a significant factor influencing perceptions of progress (*P* = 0.007). Specifically, older respondents (over 40 years) were more likely to report substantial or major progress, whereas younger participants more often perceived little or no improvement. (Table 1)

**Table 1:** Closing the gender gap by sex and age

	Gender					Age (years)				
	Female		Male		*P* value*	≤ 40		>40		*P* value*
	*n*	%	*n*	%		*n*	%	*n*	%	
**What approach should be taken in the oncology field in order to foster gender equality in the workplace** ^**b**^										
Promote worke-life balance	95	62,5	40	74,1	0,124	58	68,2	77	63.6	0,494
Development and leadership training	57	37,5	18	33,3	0,585	31	36,5	44	36,4	0,987
Offer and support flexible work	56	36,8	16	29.6	0,340	39	45,9	33	27,3	**0**,**006**
Transparent career paths and salary structures	65	42,8	14	25,9	**0.029**	33	38,8	46	38,0	0,907
**Progress made in closing the gender gap in the oncology field compared to when you started working**					0,697					**0**,**007**
No progress	19	12,9	7	13,2		15	18,3	11	9,3	
Minor progress	32	21,8	8	15,1		14	17,1	26	22,0	
Moderate progress	27	18,4	10	18,9		14	17,1	23	19,5	
Significant progress	10	6,8	7	13,2		2	2,4	15	12,7	
Major progress	6	4,1	3	5,7		1	1,2	8	6,8	
I don’t know	53	36,1	18	34,0		36	43,9	35	29,7	
**Which of the following programmes should Croatia implement to foster gender equality in oncology** ^**b**^										
Mentorship programme for female oncologists	61	40,1	12	22,2	**0.018**	32	37,6	41	33,9	0,578
Scholarship to learn from leaders in the field	53	34,9	13	24,1	0,144	29	34,1	37	30,6	0,592
Flexible educational/ fellowship programmes	59	38,8	19	35,2	0,637	36	42,4	42	34,7	0,266
Family-friendly facilities at oncology events	42	27,6	17	31,5	0,591	29	34,1	30	24,8	0,145
Online professional career development tools	31	20,4	14	25,9	0,398	20	23,5	25	20,7	0,624
Promoting gender balance through quotas in committees, faculties, and events	71	46,7	12	22,2	**0**,**002**	31	36,5	52	43,0	0,349

^a^*P* value from a chi-square test for categorical variables or a U test by ManneWhitney for continuous ones

^b^More than one answer possible

These findings reinforce the universality of challenges faced by female oncologists across Europe, while also highlighting some national differences that should be considered in tailoring future gender equity strategies.

## Discussion

Our national survey among oncology professionals in Croatia, inspired by the 2021 ESMO Women for Oncology (W4O) study, confirms key international trends while highlighting important regional distinctions that warrant targeted strategic attention. The most prominent similarity between the two studies is the persistent challenge of achieving work-life balance, identified in both surveys as the primary barrier to career advancement. In our study, however, more than 61% of respondents reported working overtime, and 86% regularly worked on weekends or public holidays, reflecting the intensity of clinical workload within the national context.

Career dissatisfaction was higher in our sample (34%) than in the ESMO cohort (28%), particularly among younger oncologists (≤ 40 years), 41% of whom reported dissatisfaction. Furthermore, 64% of participants-especially early-career professionals-felt they had not yet fulfilled their professional potential, signaling opportunities to further enhance retention, mentorship, and career development within the oncology workforce.

Unlike the 2021 ESMO findings, which highlighted the underrepresentation of women in leadership, our data emphasize administrative burden as a significant obstacle to career progression and engagement in leadership roles. Nearly 60% of respondents reported substantial administrative responsibilities, and 80% considered that administrative processes should be reorganized to allow more time for clinical and academic work. These findings provide valuable guidance for future organizational improvements.

Gender inequality remains present, perceived by 40% of respondents, although our findings align with international trends indicating a gradual reduction in gender-related barriers. Patient bias was also noted, affecting women and younger oncologists more strongly, consistent with the ESMO results. Respondents strongly supported interventions to enhance workplace equity and professional conditions, including measures to improve work-life balance (68% versus 57% in the ESMO sample), leadership training, and flexible working models. Greater support for gender quotas in Croatia compared with the ESMO sample (43% versus 19%) suggests openness toward affirmative policies as a means to enhance transparency and equal opportunities.

It is important to highlight that oncology policy in Croatia is largely guided by the National Strategy for Cancer Control 2020–2030 (NSCC) and Europe’s Beating Cancer Plan. These strategic frameworks recognize the importance of strengthening the oncology workforce and include concrete measures related to workforce planning, specialist training, development of research capacity, modernization of work organization, digitalization, and progressive reduction of administrative workload. These measures are designed to enhance professional development, improve career satisfaction, and support efficient use of oncology professionals’ time.

Croatia also actively participates in European initiatives such as EU4Health and Horizon Europe, which enhance resources for education, innovation, and professional networking. While much of these initiatives focus on improving cancer outcomes, there are substantial opportunities to align these policies with workforce needs, including training, mentorship, and leadership development. Our findings provide relevant national insight that can help guide the implementation of these policies toward areas where oncology professionals experience the greatest challenges, such as administrative burden, work-life balance, and career advancement.

A key limitation of our study is the smaller sample size (*n* = 206) compared with larger international datasets such as the 2021 ESMO W4O survey. Although the sample reflects a representative cross-section of oncology professionals in Croatia, the absence of systematic sampling may have introduced selection bias. Participants who are more aware of or affected by workplace inequities may have been more motivated to respond. While additional multivariable analyses could help identify specific factors associated with professional development barriers, our sample size did not allow for reliable execution of such analyses, particularly after stratification by demographic characteristics, family responsibilities, and workplace setting. Nevertheless, the consistency of our findings with international data supports the relevance of the observed patterns. Larger, systematically sampled, and longitudinal studies would enable a deeper understanding of national and regional workforce-specific barriers in oncology.

## Conclusion

Croatian data broadly align with pan-European trends, yet the intensity of administrative workload, extended working hours, and perceptions of professional stagnation-particularly among early-career oncologists-highlight areas warranting further attention. National and European policies provide a robust strategic framework, and our findings offer insights that may help inform their ongoing development and implementation to better support the oncology workforce. Future initiatives could consider addressing workload, systemic barriers, and career development structures to promote a sustainable, equitable, and effective oncology workforce.

## Supplementary information

Below is the link to the electronic supplementary material.

Supplementary Material 1

## Data Availability

The anonymized dataset and survey instrument are available from the corresponding author upon reasonable request.

## Abbreviations

ESMO: European Society for Medical Oncology
W4O: Women for Oncology
WHO: World Health Organization
NSCC: National Strategy for Cancer Control 2020–2030
EU4Health: European Union health programme EU4Health
